# Challenges in Implementing a Mobile AI Chatbot Intervention for Depression Among Youth on Psychiatric Waiting Lists: Randomized Controlled Study Termination Report

**DOI:** 10.2196/70960

**Published:** 2025-09-05

**Authors:** Junichi Fujita, Yuichiro Yano, Satoru Shinoda, Noriko Sho, Masaki Otsuki, Akira Suda, Mizuho Takayama, Tomoko Moroga, Hiroyuki Yamaguchi, Mio Ishii, Tomoyuki Miyazaki

**Affiliations:** 1 Department of Child Psychiatry, Yokohama City University Hospital, 3-9, Fukuura, Kanazawa-ku, Yokohama, 236-0004, Japan, 81 0457872800; 2 Psychiatric Center, Yokohama City University Medical Center, Yokohama, Japan; 3 Department of Biostatistics, Yokohama City University School of Medicine, Yokohama, Japan; 4 Kanagawa Children's Medical Center, Yokohama, Japan; 5 Fujisawa City Hospital, Fujisawa, Japan; 6 Department of Psychiatry, Yokohama City University School of Medicine, Yokohama, Japan; 7 Center for Promotion of Research and Industry-Academic Collaboration, Yokohama City University, Yokohama, Japan

**Keywords:** randomized controlled trial, AI chatbot, acceptance and commitment therapy, mental health, psychiatry, children, adolescents, Japan

## Abstract

**Background:**

The mental health of children and adolescents is a growing public health concern, with increasing rates of depression and anxiety impacting their emotional, social, and academic well-being. In Japan, access to timely psychiatric care is limited, leading to extended waiting periods that can range from 3 months to a year. Artificial intelligence (AI)–driven chatbots, such as emol (Emol Inc) that integrates acceptance and commitment therapy, show potential as digital solutions to support young patients during these waiting times. The AI chatbot emol was selected based on a comprehensive review of Japanese mental health technology apps, including in-person evaluations with company representatives.

**Objective:**

This exploratory parallel-group randomized controlled trial examined the feasibility of using an AI chatbot emol with pediatric and adolescent individuals on psychiatric waiting lists.

**Methods:**

Participants aged 12‐18 years were recruited from 4 hospitals in Kanagawa Prefecture and randomly assigned to either an intervention group, receiving 8 weekly chatbot sessions, or a control group, receiving standard mental health information. The primary outcome was the change in scores on the 9-item Patient Health Questionnaire from pre- to postintervention. Secondary assessments, such as voice and writing pressure analysis, provided additional engagement metrics, with data collected at baseline, during the intervention, and at week 9.

**Results:**

Of the 96 eligible individuals on psychiatric waiting lists, 8 expressed interest and 3 provided initial consent. However, all participants subsequently withdrew or were excluded, resulting in no evaluable data for analysis. Low engagement may have been influenced by the perceived irrelevance of digital tools, complex protocols, and privacy concerns.

**Conclusions:**

Significant barriers to engagement suggest that digital interventions may need simpler protocols and trusted environments to improve feasibility. Future studies could test these interventions in supportive settings, like schools or community centers, to enhance accessibility and participation among youth.

## Introduction

Depression and anxiety in children and adolescents are increasingly recognized as significant public health issues, profoundly affecting their emotional, social, and academic development [1,2]. Early intervention is crucial, as untreated depressive symptoms in youth are associated with a higher risk of recurrent episodes in adulthood [3]. Although traditional face-to-face mental health services have been the standard approach, research indicates that adolescents and young adults often prefer online mental health support over in-person consultations. A previous study found that young people’s preferences for mental health help-seeking vary significantly by age and stage of development, with adolescents showing a greater inclination toward online resources due to increased privacy, reduced stigma, and enhanced autonomy [4]. This preference for digital mental health support is especially relevant for those with social anxiety, depression, or concerns about confidentiality. Digital interventions may provide a more accessible entry point to mental health care for youth who might otherwise avoid seeking traditional services, potentially addressing treatment gaps during critical developmental periods.

Despite the growing demand for effective treatments, access to child and adolescent psychiatric services remains limited due to resource shortages, resulting in extended waiting periods for consultation worldwide [5]. In Japan, access to child and adolescent mental health services remains a significant challenge, with a severe shortage of trained specialists and long waiting times for consultation. Previous studies have highlighted that the mental health system is primarily focused on adult and geriatric care, leaving pediatric mental health services underdeveloped and difficult to access. The limited availability of trained professionals contributes to prolonged waiting periods for care [6]. At Yokohama City University Hospital, for example, as of October 2023, the waiting time for appointments was 8 months, with over 120 patients aged 12‐18 years on the psychiatric waiting list. The impact of the COVID-19 pandemic has further aggravated the mental health of young individuals globally, with rates of depression and anxiety among children and adolescents approximately doubling compared to prepandemic levels [7]. The increasing prevalence of adolescent depression and prolonged waiting periods for psychiatric care presents urgent challenges in mental health service delivery. For young patients on waiting lists, the lack of timely intervention can exacerbate symptoms and increase the risk of severe outcomes, such as self-harm, suicide attempts, or psychotic experiences [8,9].

Cognitive behavioral therapy (CBT), such as acceptance and commitment therapy (ACT), is a first-line treatment for depression and anxiety in young people, yet barriers such as limited availability of trained therapists highlight the need for alternative delivery methods [10]. Digital health technologies, including artificial intelligence (AI)–driven chatbots, offer an innovative approach to bridge the gap between service demand and supply. Existing studies have shown that internet-delivered CBT can be as effective as therapist-delivered CBT for treating depressive symptoms in children and adolescents [11]. Preliminary research on AI chatbots has demonstrated significant reductions in depression and anxiety among young users, underscoring the potential of these digital tools [12,13].

Despite the growing interest in digital mental health interventions, there is limited research on the effectiveness of AI chatbots specifically for children and adolescents awaiting psychiatric consultation. Although previous studies have highlighted the potential benefits of AI chatbots in reducing depressive symptoms [14], these interventions have not been systematically tested among high-need populations, such as youth on psychiatric waiting lists. Given the prevalence of depressive symptoms among pediatric psychiatric patients and the potential for severe consequences, timely intervention is crucial [7,8]. By offering mental health support through accessible digital devices like smartphones, the AI chatbot enables preconsultation care that can be implemented in various settings, including schools. This approach could facilitate early intervention and support, providing a bridge until professional care becomes available and making mental health resources more accessible to young individuals outside traditional clinical environments.

Japan has a growing number of mental health technology apps, including various AI-driven chatbots, as outlined in recent market analyses. Several AI chatbots for mental health care have demonstrated reasonable feasibility, acceptability, and potential usefulness in Japan [15,16]. However, despite the high smartphone adoption rate among young people (96.9% among those aged 18‐29 years), actual usage of health management services remains notably low at only 21.6% [17]. This gap between technology access and health-specific app usage highlights a significant opportunity for targeted digital mental health interventions. In the Japanese cultural context, health care services are evaluated through a balanced assessment where technical quality of care and health care staff behavior are both considered important factors that can compensate for each other in forming overall service quality judgments [18]. This tendency to value both technical expertise and interpersonal interactions may partially explain why Japanese patients prefer in-person medical consultations, potentially influencing the adoption rate of digital mental health interventions. Research on the social acceptance of smart health services in Japan has identified several key factors influencing adoption, including trust in service providers, perceived benefits and necessity, and risk perception regarding personal data protection [19]. These findings are particularly relevant for mental health apps where highly sensitive personal information is collected and used.

While various mental health chatbots exist in the Japanese market, few have been developed specifically for children and adolescents. For example, AI chatbot emol (Emol Inc), developed in Japan, offers structured, ACT-based interventions through an accessible, engaging interface tailored for youth [20]. By leveraging its therapeutic design and user-friendly features, it provides interim support to adolescents awaiting professional care. Despite these technological advancements, there remains a significant research gap regarding the effectiveness of AI chatbots for supporting children and adolescents on psychiatric waiting lists. Given the extended waiting periods for psychiatric consultations in Japan and the increasing prevalence of mental health challenges among youth, investigating digital interventions that can provide interim support becomes particularly important. Previous studies have shown promising results for internet-delivered cognitive behavioral therapy among young people [11], but few have specifically examined AI-driven interventions for those awaiting professional psychiatric care.

We hypothesized that using the AI chatbot would significantly improve depressive symptoms and reduce clinical symptoms among children and adolescents on psychiatric waiting lists compared to standard care, thus enhancing mental health outcomes during the waiting period. The purpose of this study was to evaluate the effectiveness of the AI chatbot in improving depressive symptoms during the waiting period for children and adolescents on waiting lists, as a preliminary phase. The primary aims were to estimate the data acquisition rate and dropout rate in the intervention group, estimate the difference in depressive symptom change between the intervention and control groups before and after the intervention, and validate case number calculations for the next randomized controlled trial (RCT).

## Methods

### Study Design

This exploratory parallel-group RCT assigned participants to either an intervention group using the AI chatbot or a control group receiving general mental health information through a publicly available website, featuring clinical information that children and their families commonly review before their appointments. Unlike “standard care,” which typically involves direct clinical assessment and treatment planning by a mental health professional, the control condition in this study only provided access to publicly available mental health education materials. No interactive therapeutic elements, personalized psychological interventions, or professional counseling were included in the control condition. This design reflects the real-world experience of many psychiatric patients on waiting lists in Japan, who often receive only basic mental health information while awaiting consultation.

Participants were centrally randomized by Nouvell Plus Inc using a minimization method based on baseline scores on the 9-item Patient Health Questionnaire (PHQ-9) scores and gender to prevent significant imbalance. Blinding was not implemented as it was deemed unnecessary for the intervention.

Recruitment materials explicitly stated that the study was conducted by researchers from Yokohama City University. This information was included on printed leaflets distributed to potential participants. The affiliation was presented to enhance credibility but may also have influenced participant expectations and willingness to enroll. Participants were briefed about the study objectives, procedures, and potential risks through an online recruitment session. Written informed consent was obtained from both participants and guardians before enrollment.

No changes to the trial methods, including eligibility criteria, were made after trial commencement.

### AI Chatbot Selection Process

To identify the most suitable AI chatbot for supporting adolescents on psychiatric waiting lists, a comprehensive evaluation of existing mental health apps in Japan was conducted. The selection process involved the following steps.

#### Comprehensive Review of Apps

Publicly available AI-driven mental health chatbots were reviewed for their therapeutic frameworks, usability, and relevance to adolescents. Special consideration was given to apps incorporating evidence-based treatments such as ACT.

#### In-Person Evaluation With Developers

Developers of shortlisted chatbots were interviewed to gain deeper insights into app features, target audiences, and implementation strategies.

#### Selection Criteria

Chatbots were evaluated based on the following criteria:

- Integration of evidence-based therapeutic frameworks (eg, ACT)
- Accessibility and ease of use for adolescents
- Engagement-focused design, including gamified elements or interactive interfaces

#### Compatibility With the Study’s Requirements for Digital Interventions in Clinical Settings

Finally, the AI chatbot emol, developed by Emol Inc [20] and released in March 2018, was selected for its integration of ACT, an evidence-based treatment for depression and anxiety. The chatbot was developed in collaboration with clinical psychologists and researchers to integrate ACT-based principles. The chatbot includes features such as conversational AI, emotional logging, interactive exercises, gamification elements, and monitoring tools to support user engagement. This therapeutic approach enables the AI chatbot emol to offer targeted, structured interventions, distinguishing it from chatbots with less comprehensive therapeutic frameworks. The user interface uses a minimalist design, with simple text-based interactions and no external hyperlinks. Content was developed in collaboration with clinical psychologists and researchers at Emol Inc, ensuring alignment with ACT-based principles. The chatbot provides asynchronous communication, allowing users to engage at their convenience without requiring real-time interaction.

The AI chatbot emol’s design prioritizes accessibility and engagement, particularly for young users, by featuring a friendly AI character named Roku. The chatbot provides asynchronous communication, allowing participants to engage at their convenience. Sessions are preprogrammed to follow a consistent instructional strategy, beginning with a mood check-in and concluding with goal-setting exercises. Additional features, such as emotional logging and sleep tracking, allow users to actively monitor their mental health. The AI chatbot emol’s comprehensive approach to digital mental self-care makes it especially well-suited for adolescents, who may prefer digital interactions over traditional therapy. By offering an approachable alternative for managing mental health challenges, emol has the potential to fill a critical gap in mental health service delivery for young people awaiting professional care.

However, no formal feasibility or usability studies have been conducted specifically for children and adolescents awaiting psychiatric consultation. The selection of emol for this study was based on a comprehensive review of Japanese mental health technology apps and direct discussions with the developers.

In this study, Emol Inc provided emol at a discounted rate. This is disclosed in the Conflicts of Interest section.

Details regarding the character design of emol and its ACT-based interactions with the character Roku are provided in Multimedia Appendix 1.

### Participants

Participants in this study were individuals on the psychiatric waiting list for Yokohama City University Hospital, Yokohama City University Medical Center, Kanagawa Children’s Medical Center, and Fujisawa City Hospital; all 4 hospitals are located in the southeastern region of Kanagawa Prefecture. These hospitals play a central role in child psychiatric services in the eastern part of Kanagawa Prefecture, Japan. Participants had to be aged between 12 and 18 years at the time of enrollment. As of January 2024, the population of 10‐ to 19-year-olds in Kanagawa Prefecture was estimated to be 774,283 [21].

The recruitment period was planned to span from October 1, 2023, to September 30, 2024, allowing sufficient time to ensure robust participant enrollment and data collection.

### Inclusion and Exclusion Criteria

The inclusion criteria were as follows: (1) a score of 10 or higher on the PHQ-9, indicating clinical depressive symptoms; (2) access to a device capable of running emol; (3) access to online interviews via Zoom (Zoom Inc); (4) proficiency in reading and writing Japanese at the upper elementary school level; and (5) agreement to abstain from using other mental self-care apps during the study period.

The exclusion criteria were as follows: (1) patients deemed to require urgent care by a child psychiatrist, (2) patients reporting a suicide attempt within the past 2 weeks on their screening questionnaire or patients in urgent need of physical treatment due to conditions such as anorexia, (3) patients who had received treatment at another psychiatric facility within the past month or who expressed a desire for future treatment at such a facility, (4) patients who were continuing to take psychotropic medications prescribed by the above-mentioned facilities, and (5) patients unable to complete diaries due to physical issues such as injury.

### Intervention Group

The intervention group received general mental health information via the Yokohama City University Child Psychiatry Department’s website, “Oyako-no Kokoro-no Tomarigi” [22], which provides short video programs and texts that provide easy-to-understand explanations of common mental health issues for children and adolescents.

In addition, 8 weekly sessions were provided by emol. Each session lasted between 20 and 30 minutes. No major bug fixes, system downtimes, content changes, or unexpected events occurred during the trial that could have influenced the intervention’s functionality or delivery. During the trial period, the version of emol remained unchanged, and no major content updates, feature modifications, or dynamic content changes occurred. The intervention was evaluated as a stable version, ensuring the replicability of study findings. While the current version reflects the intervention used in this study, future changes may occur. To ensure replicability, screenshots and videos of the interface are available upon request from the developers. Participants accessed emol via their personal smartphones. All participants needed to have a stable internet connection and a device capable of running the app. Access to the chatbot was restricted to study participants only, and no public demo mode was available. However, a free sample version of the emol program, which differs from the study version, is publicly available on the website. Participants were not required to pay any fees for accessing the chatbot during the study. A stable internet connection and a compatible device were necessary for access. No critical secular events, such as changes in internet resources or hardware requirements, occurred during the study period.

While we await specific dialogue examples from Emol Inc to further illustrate how these ACT processes were implemented conversationally, the session structure was designed to progressively build psychological flexibility through these interconnected processes. The detailed session structure and content are presented in Multimedia Appendix 2, and an example of a user interaction with the AI character Roku is illustrated in Multimedia Appendix 1.

The session overview in emol included the following:

- Session 1 (distress and enduring): introduces psychological flexibility by helping users recognize their distress patterns, aligning with the acceptance process of ACT
- Session 2 (avoidance of experiences): addresses cognitive defusion by identifying experiential avoidance patterns and encouraging users to observe their thoughts rather than becoming entangled in them
- Session 3 (control over what can be managed): emphasizes being present and distinguishing between controllable and uncontrollable aspects of experience
- Session 4 (acceptance): deepens the acceptance process by guiding users to embrace difficult emotions rather than struggling with them
- Session 5 (observing from a detached perspective): develops self-as-context by helping users adopt an observer perspective toward their experiences
- Session 6 (life values): explores the values process by assisting users in identifying what matters most to them personally
- Session 7 (commitment): focuses on committed action by translating values into concrete behavioral goals
- Session 8 (continuation of commitment): reinforces committed action while integrating all ACT processes for sustainable change

Weekly online assessments were conducted at week 0, during the intervention period, and at week 9. No additional cointerventions, training sessions, or structured support were provided beyond these assessments. For routine application outside of the RCT setting, no training is required for users. No automated prompts or reminders were used during the trial or for routine application outside of the RCT setting. The periodic assessments were administered by nonphysician research assistants, who performed minimal mental status checks and checked assessment items, such as PHQ-9, Athens Insomnia Scale (AIS), and adverse events. Simultaneously, nonphysician research assistants saved recorded audio data for voice analysis. They also provided technical assistance when required. Human support was limited to assessment and monitoring during the intervention, with no direct involvement in the therapeutic process. For routine application outside of the RCT setting, no human involvement would be required. For measuring writing pressure, participants were provided with an intelligent pen pressure device developed by Zebra Holdings Inc and given a diary in which they recorded the date, time, weather, and mood (options included “good,” “normal,” or “bad”) within 2 hours of waking. Research assistants encouraged participants to use the pen consistently for their diary entries.

### Control Group

The control group received general mental health information via the Yokohama City University child psychiatry department’s website, “Oyako-no Kokoro-no Tomarigi” [22]. This website provides educational resources about common mental health conditions in children and adolescents through easy-to-understand videos and text explanations specifically designed for young people. The video content features conversations between teddy bear and rabbit avatars discussing common mental health symptoms and concerns in children and adolescents, followed by child-friendly explanations from a child psychiatrist. Topics covered in these educational videos include suicidal thoughts, lack of energy/motivation, anxiety, isolation and loneliness, obsessive worrying, attention difficulties, self-harm behaviors, sleep problems, and auditory hallucinations. The child psychiatrist appearing in these videos is one of the authors of this study (JF). The website also contains separate sections with mental health resources for children and families, including multiple question and answer entries about children’s mental health issues. These materials are purely informational and educational in nature, rather than providing interactive or personalized therapeutic interventions. Participants were free to view the videos at their own discretion, without a predefined schedule. However, research assistants confirmed and recorded whether participants had viewed the assigned video content during each assessment session.

Unlike the intervention group, participants in the control group did not receive any structured therapeutic interaction through the AI chatbot. This design allowed for comparison between passive information provision (control) and active personalized therapeutic engagement (intervention).

Participants in the control group underwent the same regular online evaluations and diary recording process as those in the intervention group.

### Criteria for Discontinuation

Criteria outlining conditions for discontinuation of individual participants and termination of the study are mentioned in Textbox 1.

Textbox 1. **The criteria for discontinuing the intervention.**

The criteria for discontinuing the intervention for individual participants were as follows:

1. Withdrawal of consent by the participant or their legal representative.
2. Determination that the participant no longer met the inclusion criteria or met any exclusion criteria postenrollment.
3. Worsening of symptoms or findings that made study continuation challenging.
4. The occurrence of adverse events that posed challenges to study continuation.
5. Initiation of additional treatments, such as psychiatric care, counseling, or the use of mental health apps.
6. A determination by the principal investigator or sub-investigator that continuation was otherwise undesirable.

The criteria for study termination were as follows:

1. Determination that the study intervention lacked expected efficacy, posed safety concerns, or was no longer meaningful to continue.
2. Significant delays in case registration, frequent protocol deviations, or other factors made the study completion difficult.
3. Occurrence of serious compliance issues affecting study execution.

### Primary Outcome

The primary outcome was the change in PHQ-9 scores from pre- to postintervention. This measure was selected because assessing treatment efficacy through PHQ-9 score changes is commonly recommended and widely accepted in clinical research, including in Japan [23]. The PHQ-9, a self-administered questionnaire consisting of nine items, evaluates the presence and severity of depressive symptoms based on *Diagnostic and Statistical Manual of Mental Disorders, Fourth Edition* (*DSM-IV*) criteria for major depressive disorder within the past 2 weeks. The total PHQ-9 score ranges from 0 to 27, with higher scores indicating more severe depressive symptoms. The PHQ-9 score obtained at week 0 was used as the baseline.

### Secondary Outcomes

The secondary outcome measures evaluated the correlation and relationship between changes in the primary outcome measure (PHQ-9) and the following items.

#### Athens Insomnia Scale

The AIS is a self-assessment tool developed as part of the World Health Organization’s “World Project on Sleep and Health” to evaluate insomnia with high reliability and validity [24]. The scale includes 8 items, with 5 assessing nighttime sleep difficulties (“sleep onset,” “nighttime awakenings,” “early morning awakenings,” “sufficiency of total sleep duration,” and “satisfaction with sleep quality”) and 3 evaluating daytime functional impairment (“daytime mood,” “daytime activity level,” and “daytime sleepiness”). Participants rated the frequency of these experiences (at least 3 times a week in the past month) on a 4-point scale. A total score of 4 or more suggested suspected insomnia, while a score of 6 or more indicated insomnia.

#### Voice Analysis

During online consultations, participants’ voices were recorded while they conversed with the interviewer. Voice data was analyzed by SHIN4NY Inc, with recordings provided to the company for analysis. A previous study demonstrated that a speech emotion recognition model could predict depression [25].

#### Writing Pressure Analysis

Upon providing consent, participants received a diary and instructions on how to record entries using an intelligent pen pressure device developed by ZEBRA HOLDINGS Inc. This device, a mechanical pencil, automatically captured data on writing pressure, acceleration, and pen angle, storing it without requiring additional action from the participant. Previous research has suggested that an intelligent pen capable of measuring writing pressure may predict anxiety levels [26].

These data were collected at baseline (week 0), during the intervention, and at the study’s conclusion (week 9) through online consultations. Evaluations included PHQ-9, AIS, voice analysis, and intelligent pen pressure device data.

### Data Management

Data entry was conducted by research assistants and verified by the principal investigator. A data dictionary guided the coding process, and central monitoring took place once during the study period based on the collected case report forms.

This study managed data for both clinical outcomes and user engagement. In addition to the primary and secondary outcome measures, engagement metrics were tracked using CSV data logs provided by Emol Inc, including (1) total usage time, (2) average daily usage time, (3) usage time periods, (4) last completed session, (5) last usage date, (6) first usage date, and (7) session progression history. The purpose of analyzing these engagement metrics was 2-fold. First, we aimed to identify usage patterns and their potential relationship with changes in depressive symptoms. Second, these data were intended to assess whether participants engaged appropriately with the app and to evaluate whether they met the expected level of engagement for effective intervention. For example, adequate engagement might be defined as completing a minimum number of chatbot sessions or maintaining consistent interaction over time.

### Statistical Analysis

The analysis populations were defined as follows, with the primary analysis population being the full analysis set (FAS). This study included all participants who were registered, randomized, received at least one session of the trial intervention, and had available efficacy data. Participants were excluded if baseline data were not obtained or if significant protocol violations occurred. In addition, the FAS included participants who had no major deviations from the study protocol. Missing data were analyzed as observed without imputation, with the primary analysis performed on the FAS. As this study was exploratory in nature and lacked previous research in this specific population, no imputation for missing data was conducted.

The target sample size for the study was 60 participants (30 in each group). This calculation assumed an expected between-group mean difference in PHQ-9 change scores of 7, an SD of 10, a 2-sided significance level of .10, and a power of 0.80, using an independent 2-sample *t* test. The estimated sample size was 26 participants per group, and a target of 30 participants per group was set to allow for possible exclusions.

The primary analysis was conducted using the FAS. Changes in PHQ-9 scores from pre- to postintervention were presented as mean (SD). An analysis of covariance estimated the difference between groups, including the 90% CI and *P* value for the group comparison. A 2-sided test was used, with statistical significance determined at *P*<.10. The secondary analysis was conducted on the per-protocol set using the same summary and analytical methods as the primary analysis.

### Protocol Version and Amendments

Protocol version 1 was initially approved on September 27, 2023, with version 2 subsequently approved on February 15, 2024.

### Ethical Considerations

This study was registered with the Japan Registry of Clinical Trials under registration number jRCT1032230427. The full trial protocol is available on the Japan Registry of Clinical Trials website [27]. Approval for the study was granted by the Ethics Committee of Yokohama City University (approval F230907001). All procedures were conducted in accordance with the ethical standards outlined in the Declaration of Helsinki and the “Ethical Guidelines for Medical and Health Research Involving Human Participants.” Written informed consent was obtained from both participants and their guardians before enrollment. When possible, assent from participants was also sought; if direct assent was difficult to obtain, informed assent was acquired as an alternative. If participants exhibited severe psychological distress or suicidal ideation during the study, the research team had a protocol in place to guide them to appropriate psychiatric services. Emergency contact information for crisis intervention was provided to all participants and their guardians. The study adhered to safety monitoring procedures to ensure participant well-being throughout the intervention. Participant privacy and confidentiality were strictly protected, and collected data were used solely for research purposes. All personal information was handled in accordance with Yokohama City University’s privacy policy [28]. Findings from the study are to be disseminated through peer-reviewed journals and academic conferences.

## Results

This study was conducted across 4 hospitals in Kanagawa Prefecture: Kanagawa Children’s Medical Center, Fujisawa City Hospital, Yokohama City University Hospital, and Yokohama City University Medical Center, targeting pediatric and adolescent psychiatric patients awaiting consultation. Recruitment materials were distributed from October 2023 to June 2024 to patients on waiting lists at each hospital as follows:

- Kanagawa Children’s Medical Center: 78 patients
- Fujisawa City Hospital: 8 patients
- Yokohama City University Hospital: 10 patients
- Yokohama City University Medical Center: 0 patients

A total of 96 patients received study invitations (78 from Kanagawa Children’s Medical Center, 8 from Fujisawa City Hospital, and 10 from Yokohama City University Hospital). Of these, 8 patients expressed interest by contacting us for additional information. Out of those who expressed interest, 3 patients scheduled and completed the informed consent process, while the remaining 5 did not proceed with informed consent due to a lack of response when attempting to arrange a schedule.

Among the 3 patients who completed the informed consent process, 1 participant (a female adolescent) provided consent but subsequently withdrew from the study. The participant’s family initially contacted the research team on the scheduled day of the first online session, stating:

*This morning, she became panic-stricken and is now unable to participate. Although it is the day of the appointment, would it be possible to cancel? I sincerely apologize for the inconvenience caused after all your preparations.*

In a follow-up message, the family elaborated:

*She expressed anxiety about the online interview, making it impossible to proceed. We had hoped that engaging in this activity might help her develop a more positive outlook, but perhaps it was still too challenging for her.*

**Figure 1.**
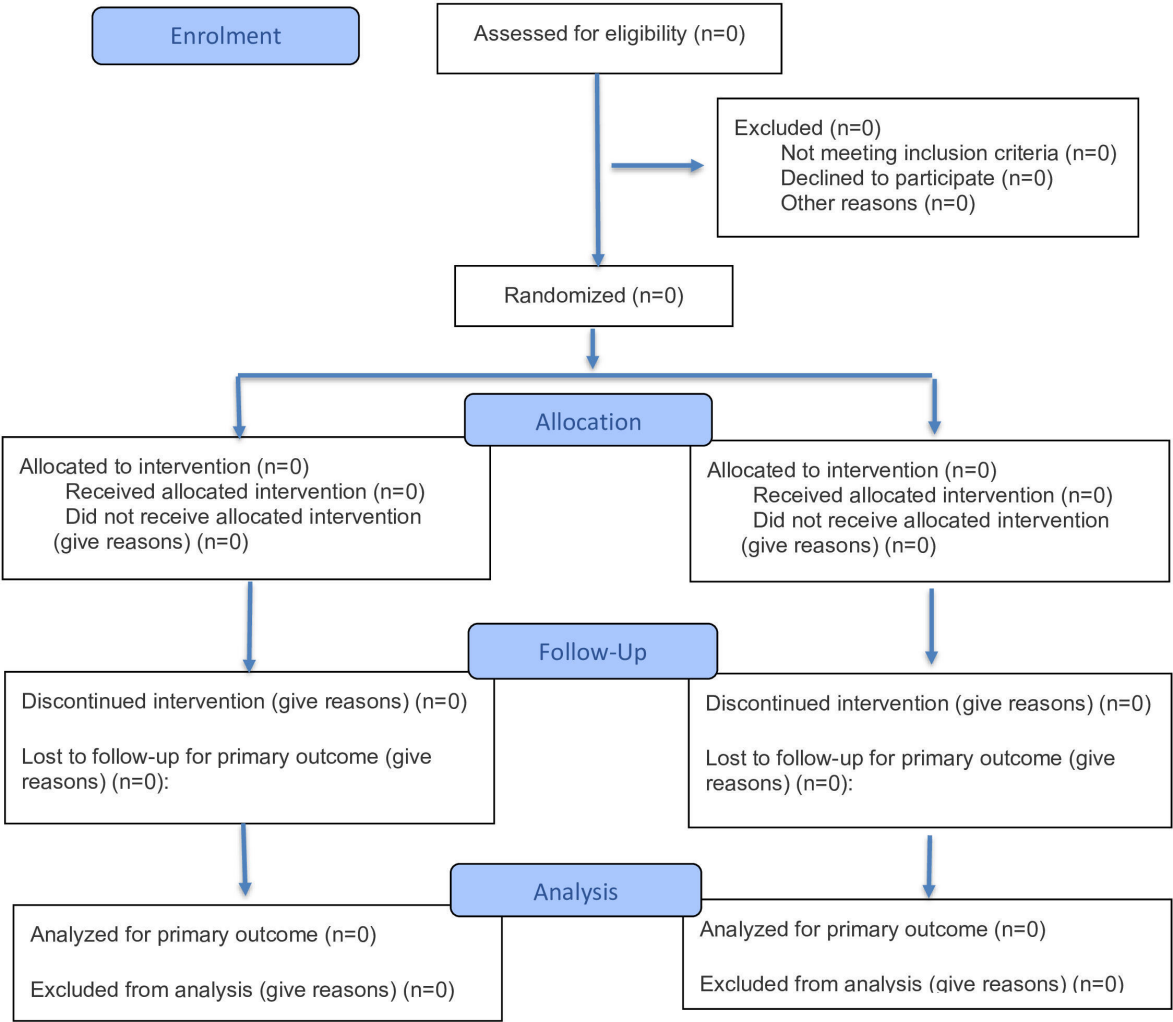
CONSORT (Consolidated Standards of Reporting Trials) 2025 flow diagram.

The other 2 patients who completed the informed consent process either declined participation due to concerns about diary recording requirements or were excluded after beginning medication at another facility. Another patient declined participation due to concerns about diary recording, and the third patient was excluded after beginning medication at another facility. Consequently, no evaluable data were obtained in this study (Figure 1).

## Discussion

### Barriers to Engagement in Digital Interventions

The primary finding of this study is that children and adolescents with mental health challenges and their caregivers showed limited interest in an AI chatbot-based intervention while awaiting psychiatric consultation, highlighting complex barriers to the adoption of digital mental health interventions. Several factors contributed to this low engagement. In this study, only one participant consented but subsequently withdrew due to a worsening of symptoms. This outcome suggests that patients with more severe symptoms may find digital interventions less suitable or accessible. However, given the need for timely intervention among high-severity cases, it is worth exploring ways to make AI-driven tools like chatbots more adaptable to their needs. Improving the usability and support level of digital tools for more severe cases could provide valuable early intervention options where in-person resources are limited.

### Digital Intervention Challenges: Accessibility, Family Influence, and Psychological Burden

First, the one participant who withdrew provides valuable insights into digital intervention barriers. Despite digital tools being promoted as accessible for those with social anxiety, this case reveals that even virtual interactions can trigger a significant psychological burden in adolescents with severe mental health challenges. The mother’s hope that “engaging in this activity might help her develop a more positive outlook” contrasted with her realization that “perhaps it was still too challenging for her,” highlighting the gap between theoretical accessibility and practical barriers. The child psychiatrist who interviewed the patient and family during adverse event verification determined that the patient experienced increased subjective burden at the time of study participation, along with anxiety and avoidance symptoms exacerbated by her underlying depressive condition. This case demonstrates a “digital intervention paradox”—while technology aims to increase accessibility, implementation requirements like scheduled online sessions can create new barriers for the very individuals they intend to help. Future interventions might need truly asynchronous options that minimize direct interaction while maintaining efficacy and safety monitoring.

Many young patients, particularly those who have not received a formal diagnosis or treatment, may struggle to understand the relevance of app-based mental health support and may not fully appreciate its therapeutic value, especially when compared to the immediate effects associated with in-person interventions like pharmacotherapy or face-to-face therapy. A previous study with small sample sizes has shown that AI chatbot app dropouts for psychogenic premature ejaculation were only around 25% among Japanese adults [16]. On the other hand, previous studies suggest that young patients often lack intrinsic motivation to engage with mental health apps, especially when their symptoms are severe enough to require professional care [29,30]. In particular, previous research indicates that individuals with more severe symptoms may benefit from more personalized support to enhance engagement and adherence to digital interventions [31]. Without a clear understanding of the intervention or trust in its benefits, these young patients may lack motivation to use the app consistently, contributing to low engagement in the study.

### Study Protocol Complexity and Privacy Concerns

This study may have unintentionally targeted a population less receptive to alternative digital interventions. Families who had already secured an upcoming psychiatric appointment may have seen little value in participating in a study involving digital interventions, preferring instead to wait for their scheduled in-person consultation. For these families, traditional in-person care may have appeared more reassuring, especially given the severity of the patient’s symptoms. Previous research on social influences in mental health service-seeking behavior among young people suggests that family is often the primary influence in choosing in-person services, whereas young people themselves tend to make decisions regarding online services [4]. Another study has also found that parents often seek informal support for their children’s mental health concerns initially, only turning to professional services as issues become more severe [32]. In addition, patients with severe symptoms or their families often prefer in-person consultations over digital interventions, perceiving in-person care as more reliable and suitable for managing serious symptoms [33]. Therefore, patients and families may value the familiarity and perceived efficacy of traditional, in-person care as a more reliable or reassuring option compared to digital alternatives. This preference likely contributed to the reluctance toward digital solutions observed in this study. Engaging patients and families earlier in the mental health care process—before they have secured traditional clinical appointments—might improve receptiveness to digital options. While an active control group could have offered a more rigorous comparison, we selected a passive control condition due to practical constraints. At the time of study planning, emol was the only adolescent-appropriate AI chatbot in Japan that integrated evidence-based psychological content (ACT), had a suitable user interface, and was available for research use. No other comparable tool was identified. Thus, we chose a passive control to reflect the real-world conditions in Japan, where patients on psychiatric waiting lists typically receive only basic informational support.

Furthermore, our study protocol involved multiple evaluation sessions, which could have imposed additional stress on participants, particularly those with social anxiety or other issues related to interpersonal interactions. Previous studies indicate that such requirements can increase dropout rates, especially among adolescents with social phobia [34,35]. In this context, the protocol’s demands may have discouraged participation and contributed to low engagement. Although efforts were made to reduce participant burden by conducting interviews online, the process of obtaining informed consent and providing detailed study information likely remained a challenge for some participants, especially those who may be sensitive to social interactions. Moreover, secondary assessment methods like voice and writing pressure analysis may have caused some participants to feel self-conscious or concerned about privacy, potentially exacerbating symptoms or causing reluctance to participate [36]. Privacy concerns are common with mental health apps, especially when personal data is analyzed, and this may affect user engagement [37]. In this study, participants may have felt uneasy about the voice recordings or writing pressure data being analyzed, as well as discussing their mental health status with researchers. These issues may have created additional barriers to engagement, particularly for young people unfamiliar with research environments. In the current study design, the exclusion of severe cases and the online interview assessment to reduce the burden of face-to-face implementation may not have been sufficient.

### Strengths and Limitations

This study had both methodological strengths and limitations. Conducting an RCT in an understudied population—children and adolescents on psychiatric waiting lists—provided valuable insights into a critical phase of mental health service delivery, and the exploratory nature of the trial allowed for close examination of data acquisition and dropout rates. In addition, our use of multiple assessment methods enabled a comprehensive evaluation of potential therapeutic effects and engagement patterns, which is rare in digital mental health studies involving youth.

However, the intensity of the intervention, including frequent evaluations and a structured protocol, may have been overwhelming for participants. This study suggests that less rigid protocols with fewer demands could enhance engagement. In addition, recruiting patients already awaiting traditional psychiatric care may have reduced openness to digital alternatives, thus limiting the generalizability of our findings. Targeting a population that has not yet committed to in-person care—such as students in school counseling or those referred by community organizations—may address this issue in future studies. Finally, while secondary measures such as voice and writing pressure analysis provide valuable data, they may also create privacy concerns that deter participation, particularly among younger users. In addition, typical limitations of eHealth trials should be noted. Participants were not blinded, which may have introduced performance bias. The informed consent process could have influenced their expectations, and the multiple outcomes planned for the study could have increased the risk of type 1 errors. Future studies should address these biases and explore strategies to improve participant engagement. Furthermore, a key limitation of this study was the lack of a structured plan for conducting qualitative interviews and analyses on dropout reasons. This was primarily because the initial study design focused on quantitative outcome measures, and the feasibility of integrating additional qualitative assessments was not fully considered. While we identified some possible factors, such as social anxiety, depressive symptoms, and difficulties with maintaining engagement, a more systematic approach to understanding participants’ experiences and challenges would have provided deeper insights.

Future studies should incorporate structured qualitative methods, such as exit interviews or surveys, to better understand engagement barriers and develop targeted strategies for improving retention and adherence in digital mental health interventions for adolescents on psychiatric waiting lists. Previous qualitative studies have identified key factors influencing user engagement, including personalization, trust in AI, and perceived relevance of content [38]. Integrating these insights into future chatbot interventions may enhance usability and acceptability.

### Conclusions

To better understand the potential of AI chatbot interventions like emol, future studies could test the app in environments where supportive, pre-existing relationships exist, such as schools or community youth centers. Conducting trials in these familiar settings may foster trust, encourage participation, and enhance data validity, ultimately increasing the accessibility and effectiveness of digital mental health interventions for younger adults.

## Supplementary material

10.2196/70960 Multimedia Appendix 1 Screenshots of the artificial intelligence chatbot emol interface.

10.2196/70960 Multimedia Appendix 2 Session structure of the AI chatbot emol aligned with Acceptance and Commitment Therapy core processes.

10.2196/70960 Checklist 1 CONSORT-EHEALTH checklist (V 1.6.1).

## Abbreviations

ACT: acceptance and commitment therapy
AI: artificial intelligence
AIS: Athens Insomnia Scale
CBT: cognitive behavioral therapy
*DSM-IV*: *Diagnostic and Statistical Manual of Mental Disorders, Fourth Edition*
FAS: full analysis set
PHQ-9: 9-item Patient Health Questionnaire
RCT: randomized controlled trial
