# Supplementary figures and images for: Challenges in Implementing a Mobile AI Chatbot Intervention for Depression Among Youth on Psychiatric Waiting Lists: Randomized Controlled Study Termination Report
Source: JMIRx Med. 2025 Sep 5;6:e70960. doi: 10.2196/70960 (PMC12413189; doi:10.2196/70960)

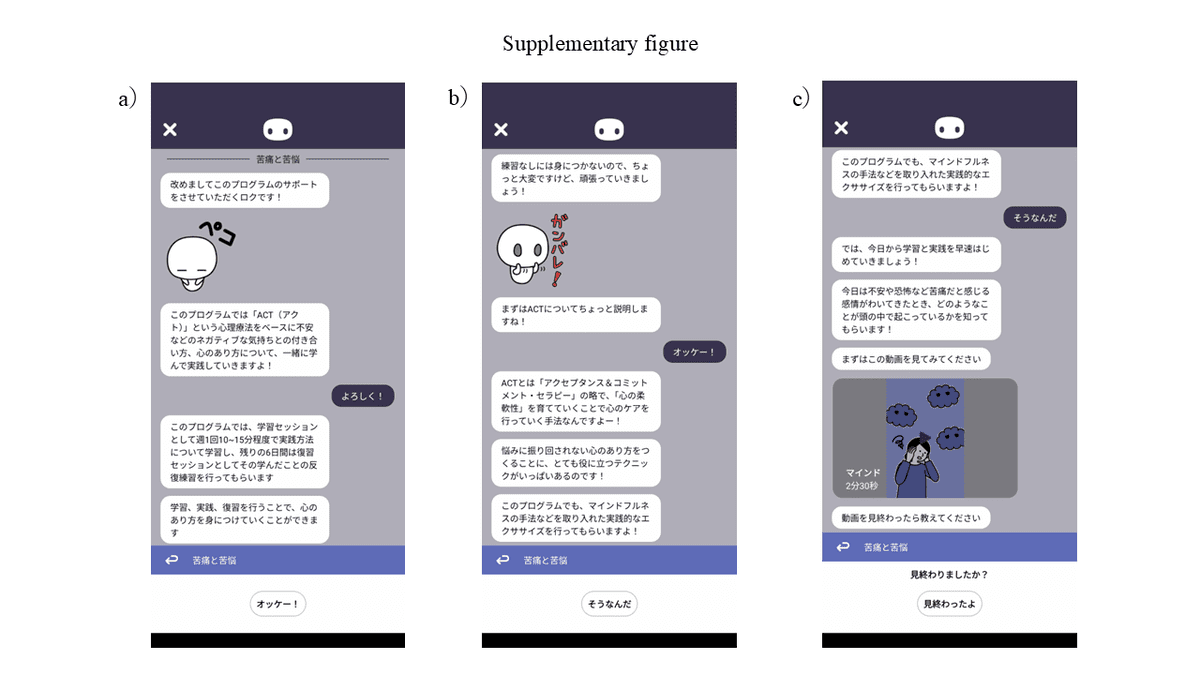

Supplement: Multimedia Appendix 1 [file xmed-v6-e70960-s001.png]
