# Supplementary material for: Challenges in Implementing a Mobile AI Chatbot Intervention for Depression Among Youth on Psychiatric Waiting Lists: Randomized Controlled Study Termination Report
Source: JMIRx Med. 2025 Sep 5;6:e70960. doi: 10.2196/70960 (PMC12413189; doi:10.2196/70960)
Supplement: Multimedia Appendix 2 [file xmed-v6-e70960-s002.docx]

**Supplementary table**

| **Session** | **Title** | **Summary of Contents** | **Duration** |
| --- | --- | --- | --- |
| Session 1 | Distress and Suffering | • Introduction to ACT  • Role of anxiety  • Examining attempts to eliminate anxiety  • Explanation of awareness exercises | 15 min |
| Review 1 | Session 1 Review Session | Review of session 1 content | 8 min |
| Session 2 | Experiential Avoidance | • Examining avoidance patterns  • Recognizing the costs of avoidance  • Creative hopelessness  • "Feeding the anxiety trap" metaphor  • Observing anxiety rather than struggling with it  • Homework: "Things I've given up due to anxiety" | 18 min |
| Review 2 | Session 2 Review Session | Review of session 2 content | 12 min |
| Session 3 | What Can Be Controlled | • Control as the problem, not the solution  • "Tug of war with monster" metaphor  • Distinguishing between controllable and uncontrollable aspects  • Considering value-aligned actions  • Homework explanation | 15 min |
| Review 3 | Session 3 Review Session | Review of session 3 content | 10 min |
| Session 4 | Acceptance | • Acceptance and willingness  • Methods for facing uncomfortable emotions  • Exercise for accepting uncomfortable emotions | 18 min |
| Review 4 | Session 4 Review Session | Review of session 4 content | 13 min |
| Session 5 | Observer Perspective | • Volleyball metaphor for thoughts and feelings about anxiety | 10 min |
| Review 5 | Session 5 Review Session | Review of session 5 content | 5 min |
| Session 6 | Life Values | • Creating a values worksheet  • Life compass | 10-20 min |
| Review 6 | Session 6 Review Session | Review of session 6 content | 5-15 min |
| Session 7 | Commitment | • Willingness  • Creating a goal achievement table | 10-20 min |
| Review 7 | Session 7 Review Session | Review of session 7 content | 5-15 min |
| Session 8 | Continuing Commitment | • Activity verification  • Strategies for when things don't go well  • Summary | 5-15 min |
| Daily Training | Daily Training) | Same content as Session 7 review | 5-15 min |
| Reflection Session | Reflection Session | Same content as Session 8 "Continuing Commitment" | 5-15 min |
